# Consumption in General Practice

**Published:** 1913-03

**Authors:** 


**Consumption in General Practice.** By H. Hyslop Thomson, M.D., D.P.H. Pp. xv, 335. London: Oxford Medical Publications. 1912. Price, 12s. 6d. net.

The second edition of this book has been entirely rewritten, and much new matter has been added. The work is divided into three sections, under the headings—Diagnosis, Prognosis and Treatment.

Of the first three chapters of Section I, dealing with the importance of early diagnosis, and the means by which it may be made, it would be difficult to speak too highly.

Those to whose lot it falls to go into the "histories" of a large number of phthisical patients know how much need there still is of a more widespread knowledge of the clinical facts taught in these chapters.

Unfortunately, the author is one of those who still cherish the bugbear that the rectal taking of temperatures " except in hospitals and sanatoria is more or less impracticable " (*sic*).

Without entering into the discussion of this question, it may be said that throughout the book those who have convinced themselves of the necessity of taking rectal observations will find many reasons to congratulate themselves on having formed this conviction.

It is strange to find in a volume which, on the whole, covers the ground most thoroughly, no mention of the variations in temperature associated with the menstrual cycle both in normal and tuberculous women.

The question of artificial pneumo-thorax is quite insufficiently dealt with.

The whole of Section II, on " Prognosis," is excellent, especially the chapters on " Indications from Localising Symptoms," and those on " Ultimate Prognosis " and " Conclusions."

The chapters on " Modern Sanatorium Treatment " and " Treatment by Auto-inoculation " are very good indeed.

Dr Thomson makes some very interesting observations from his personal experience. He has found that incontinence of urine in the female is of bad prognostic import, and also that the development of freckles in a certain type of patient with pale complexion, fair skin, and light or auburn hair often points to a very refractory type of disease.

Considerable importance in prognosis is attached to the presence of a positive diazo-reaction.

The question of the use of tuberculin in treatment is not widely entered upon, but, which is more important, the author gives fully his own methods, opinions and results.

He believes in tuberculin, sub-cutaneously, as a diagnostic agent, but looks for a marked reaction with malaise, etc. He is not apparently troubled by thoughts of induced susceptibility or reaction due to a healed focus.

It is pointed out, as it cannot be too often, that the point is not whether a " normal " person, nor even a person with a focus of inactive tuberculosis, *will* react, but whether a reaction in a patient *suspected for other reasons* is of diagnostic value.

Unlike most authorities, the writer considers hæmoptysis a contra-indication to the use of tuberculin, and he tells us nothing of his views on its use as febrifuge or in symptomatic treatment in advanced cases.

The opinion is advanced that it is dangerous to break off suddenly either treatment by induced auto-inoculations or a course of tuberculin, presumably from fear of a sudden drop in tolerance not accompanied by an equivalent drop in sensitiveness: it is not quite clear how "tapering off" with smaller doses after attaining a maximum will help in this matter.

The writer is undoubtedly right when he says that many patients who have reached a point at which they cease to inoculate themselves by exercise need then a course of tuberculin treatment.

The chapter on the treatment of symptoms and complications is very good indeed. Dr. Thomson, who has known how to take full advantage of his clinical experience, is much to be congratulated on this book.

